# Curcumin as an Enhancer of Therapeutic Efficiency of Chemotherapy Drugs in Breast Cancer

**DOI:** 10.3390/ijms23042144

**Published:** 2022-02-15

**Authors:** Reyhaneh Farghadani, Rakesh Naidu

**Affiliations:** Jeffrey Cheah School of Medicine and Health Sciences, Monash University Malaysia, Jalan Lagoon Selatan, Bandar Sunway, Subang Jaya 47500, Selangor Darul Ehsan, Malaysia

**Keywords:** drug resistance, combination therapy, cancer drug discovery, curcumin, signaling pathway, clinical trial, chemosensitizer, anticancer agent

## Abstract

Female breast cancer is the world’s most prevalent cancer in 2020. Chemotherapy still remains a backbone in breast cancer therapy and is crucial in advanced and metastatic breast cancer treatment. The clinical efficiency of chemotherapy regimens is limited due to tumor heterogeneity, chemoresistance, and side effects. Chemotherapeutic drug combinations with natural products hold great promise for enhancing their anticancer efficacy. Curcumin is an ideal chemopreventive and chemotherapy agent owning to its multitargeting function on various regulatory molecules, key signaling pathways, and pharmacological safety. This review aimed to elucidate the potential role of curcumin in enhancing the efficacy of doxorubicin, paclitaxel, 5-fluorouracil, and cisplatin via combinational therapy. Additionally, the molecular mechanisms underlying the chemosensitizing activity of these combinations have been addressed. Overall, based on the promising therapeutic potential of curcumin in combination with conventional chemotherapy drugs, curcumin is of considerable value to develop as an adjunct for combination chemotherapy with current drugs to treat breast cancer. Furthermore, this topic may provide the frameworks for the future research direction of curcumin–chemotherapy combination studies and may benefit in the development of a novel therapeutic strategy to maximize the clinical efficacy of anticancer drugs while minimizing their side effects in the future breast cancer treatment.

## 1. Introduction

Cancer is still a severe life-threatening disease that ranks as a leading cause of death around the world. The global cancer burden is rapidly rising worldwide with 19.3 million new cancer cases and about 10 million deaths in 2020, and expected to reach 28.4 million cases in 2040, a 47% increase from 2020. With the diagnosis of about 2.3 million new cases (11.7% of all cancer cases), female breast cancer has surpassed lung cancer, making it the world’s most prevalent cancer in 2020. Among women, breast cancer stands first in terms of incidence (~25%) and mortality (16%) rates in 2020 worldwide [1,2]. The variations observed in breast cancer epidemiology have revealed high incidence and low mortality rates in developed countries and low incidence and high mortality rates in developing countries. In addition, the five-year survival rate of breast cancer after diagnosis varies worldwide, with the highest rate in high-income countries (more than 90%) compared with India and South Africa with 66% and 40%, respectively, which is correlated with early detection and treatment of breast cancer [2,3,4].

**Figure 1:**
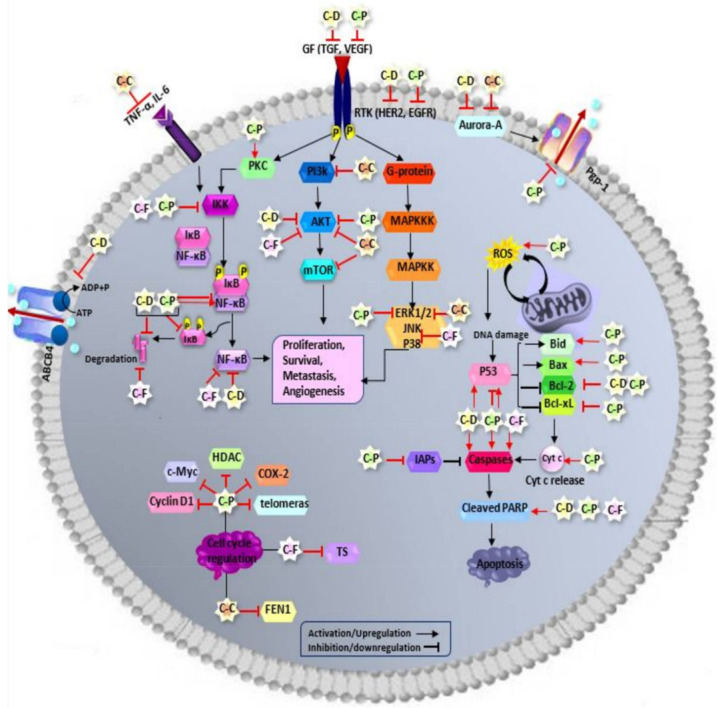
Schematic diagram of curcumin combination chemotherapy mechanism of action in breast cancer therapy. Curcumin, in combination with conventional chemotherapy drugs (doxorubicin, paclitaxel, 5-fluorouracil, and cisplatin), has enhanced their therapeutic anticancer efficacy through targeting various molecules and subsequently regulating key signaling pathways and mechanisms involved in breast cancer progression and chemoresistance. CUR—curcumin; DOXO—doxorubicin; PTX—paclitaxel; 5FU—5-fluorouracil; CIS—cisplatin, TNBC—triple negative breast cancer; EMT:—epithelial-mesenchymal transition; GSK3β—glycogen synthase kinase 3 beta; Dvl—disheveled; PTEN—phosphatase and tensin homologue; CDKs—cyclin dependent kinases; MMP-9—matrix metallopeptidase 9; ICAM-1—Intercellular adhesion molecule-1; ALDH-1—aldehyde dehrogenase-1; CSCs—cancer stem cells; EAC—Ehrlich ascites carcinoma; FEN1—Flap endonuclease1; DDP—cis-diammedichloroplatinum; PPAR-γ—peroxisome proliferator-activated receptor gamma; BDNF—brain-derived neurotrophic factor; TNF-α—tumor necrosis factor alpha; IL—interleukin; CCAT1—colon cancer associated transcript 1; C-C:—curcumin-cisplatin; C–D—curcumin-doxorubicin; C-P—curcumin-paclitaxel; C-F—curcumin-5-fluorouracil; PI3K—phosphatidylinositol-3-kinase; AKT or PKB—protein kinase B; mTOR—mammalian target of rapamycin; RTK—receptor tyrosine kinase; P—phosphorous; GF—growth factor; EGFR—epidermal growth factor receptor; HER2—human epidermal growth factor receptor 2; TGF—transforming growth factor; VEGF—vascular endothelial growth factor; TNF-α—tumor necrosis factor-alpha; IL-6—interleukin 6—Raf: rapidly accelerated fibrosarcoma; MAPK—mitogen-activated protein kinase; MAPKKK—MAPK kinase kinase; MAPKK— MAPK kinase; JNK—c-Jun N-terminal kinase; ERK—extracellular signal-regulated kinase; PKC—protein kinase C; NF-κB—nuclear factor-kappa B; IKK—inhibitor of kappa B kinase; IκB—inhibitor of NF-κB; Bcl-2—B-cell lymphoma 2; Bid—BH3 interacting-domain death agonist; Bax—Bcl-2 associated X protein; Bcl-xL—B-cell lymphoma-extra-large; Cyto c—cytochrome c; PARP—poly (ADP-ribose) polymerase; IAPs—Inhibitors of apoptosis proteins; ROS—reactive oxygen species; TS—thymidylate synthase; FEN1—Flap endonuclease 1; HDAC—histone deacetylase; COX-2—cyclooxygenase-2, ABCB4—ATP binding cassette subfamily B member 4; Pgp-1 or ABCB2—P-glycoprotein 1 or ATP-binding cassette subfamily B member 1.

In order to manage and treat breast cancer, surgical resection, radiation, and systemic therapy consisting of endocrine/hormonal therapy, chemotherapy, targeted therapy, or any combination of these approaches have been applied in breast cancer patients [2,5]. Chemotherapy is a treatment with anticancer drugs, having cancer cell killing properties, which is given intravenously or as a pill and prescribed according to patients’ performance status and pathological factors such as tumor stage, hormone receptor status, and HER2 expression. In order to manage breast cancer, chemotherapy is used either as neoadjuvant or adjuvant therapy to shrink a tumor, prevent the recurrence of breast cancer, and spread to other parts of the body prior to or followed by surgery and radiotherapy. Therefore, chemotherapy still remains as a backbone in breast cancer therapy, and its role even becomes more crucial when the patients are facing advanced and metastatic breast cancer [6,7,8,9]. Besides, it is the only systemic modality available for triple-negative breast cancer patients (TNBC) in which tumor cells do not express the estrogen receptor, progesterone receptor, and lack HER2 overexpression [10,11]. Chemotherapy works by attacking fast-growing cells in the body, including cancer cells. However, it also destroys rapidly dividing healthy cells such as cells in hair follicles, nails, mouth, digestive tract, and bone marrow involved in blood cells production. Therefore, chemotherapy-associated side effects and toxicity may include lowered resistance to infections, weakness, nausea, vomiting, hair loss, and others [12,13]. Moreover, drug resistance and tumor heterogeneity are among major problems to successful breast cancer chemotherapy [14,15,16]. In order to overcome the obstacles of current clinical drugs, many efforts have been made to enhance the chemotherapeutic impact of drugs while reducing their doses and toxic side effects in the treatment of breast cancer. In this regard, the combination of chemotherapy with natural products, including curcumin, has been of great interest to boost their anticancer efficacy. Therefore, this review aimed to discuss the promising role of curcumin in enhancing the efficacy of the most common chemotherapy drugs with a focus on doxorubicin, paclitaxel, 5-fluorouracil, and cisplatin, having diverse mechanisms of action, via combination treatment in breast cancer therapy. In addition, the possible molecular mechanisms underlying the chemosensitizing activity of curcumin and overcoming drug resistance have also been addressed here and are summarized in Figure 1. In the current review, MCF-7 and T47D cell lines are categorized in luminal A (ER+, PR+/–, HER2–), MDA-MB-231, MDA-MB-435, and BT-20 cell lines are categorized in TNBC (ER-, PR-, HER2-), while SKBR-3 cell line is categorized in HER2 positive (ER-, PR-, HER2+) breast cancer subtype.

## 2. Curcumin

Curcumin (CUR) is a secondary metabolite and bioactive component of the turmeric spice, which is obtained from the ground rhizome of the *Curcuma longa* plant, a member of the ginger family Zingiberaceae [17]. This complex molecule, having a broad range of pharmacological activities, for instance, anti-inflammatory, antioxidant, antibacterial, and anticancer properties, has been widely applied in Indian traditional medicine to prevent and treat various disorders [18,19]. Considerable evidence exists demonstrating the promising role of CUR in breast cancer therapy. CUR exerts anti breast cancer impact through targeting various regulatory proteins, including those of kinases, transcription factors, receptors, enzymes, growth factors, cell cycle, and apoptosis-related molecules, as well as miroRNAs. It has also been shown to modulate a variety of key signaling pathways of JAK/STAT, NF-ĸB, Wnt/β-catenin, PI3K/Akt/mTOR, MAPK, apoptosis, and cell cycle pathways involved in breast cancer progression and development [20,21,22,23]. 

CUR targets JAK/STAT pathway through suppression of pathway activating molecule IL-6, downregulation/inactivation of various members of JAK and STAT proteins such as JAK2, STAT3, and STAT5, inhibition of STAT translocation to the nucleus, and eventually attenuation of downstream targets of c-jun, vimentin, c-myc, snail, etc. [24,25,26]. In addition, the mechanism of CUR inhibitory effect on NF-ĸB pathway has been suggested to operate through suppression of NF-κB expression and its translocation to the nucleus, inhibition of IKK activity, and IκB degradation, which result in diminishing its target expression, cyclin D1, IAP, surviving, EMT markers, etc., in breast cancer studies [27,28,29,30]. Furthermore, CUR demonstrated the multiple suppressive effects on Wnt/β-catenin signaling components, which include inhibition of GSK3β phosphorylation, downregulation and modification of subcellular localization of β-catenin, cyclin D1, slug, and Dvl proteins leading to modulation of its downstream targets involved in metastasis and cancer stem cell activity [31,32,33]. Moreover, CUR interferes with the PI3K/Akt/mTOR pathway through its regulatory role on key molecule players of AKT, PTEN, HER2, and mTOR, which may facilitate the inhibition of cellular growth, invasion, and metastasis in breast cancer [34,35,36]. Besides, evidence strongly implicates that antiproliferative, antimigratory, and anti-invasion properties of curcumin are mediated through CUR-altered functions or expression levels of TGF, EGFR, ERK1/2, MKK4, JNK, and P38 as key components of the MAPK signaling pathway [37,38,39,40]. Along with various cell signaling pathways, the potential anticancer activity of curcumin has also correlated with promoting apoptotic cell death via altering the expression of initiator and effector caspases, PARP, antiapoptotic and proapoptotic members of the BCL-2 protein family, IAPs, miRNAs, and ROS generation [41,42,43,44]. CUR also can trigger growth arrest via targeting cyclin, CDKs, CDK inhibitors, and DNA repair proteins involved in cell cycle progression and regulation [45,46,47]. Therefore, the multitarget function of CUR, as an advantage over conventional chemotherapeutic drugs, leads to interference with different phases of breast cancer development consisting of tumor initiation and progression, invasion, metastases, and angiogenesis. Furthermore, CUR is considered a safe, nontoxic phytochemical [48,49]. 

## 3. The Combination of Curcumin and Chemotherapy Drugs in Breast Cancer Therapy

Although chemotherapeutic drugs play a vital role in breast cancer control and treatment, their effectiveness is often limited due to severe toxic side effects and the development of chemoresistance over time in breast cancer patients. In order to overcome these issues, the focus of researchers and clinicians has shifted towards cotreatment of conventional chemotherapeutic with natural products, including CUR, to enhance the efficacy of drug treatment via synergistic antitumor effect.

### 3.1. Curcumin Enhances the Efficacy of Doxorubicin in Breast Cancer Therapy

Doxorubicin (DOXO), commercially known as Adriamycin, is an antibiotic, anticancer drug, first extracted from Streptomyces bacteria in the 1970′s and classified in anthracycline group [50]. DOXO mainly interacts with DNA molecules through intercalating between base pairs in the DNA helix. This intercalation with DNA inhibits the progression of topoisomerase II, an enzyme that relaxes supercoils in DNA via breaking and rejoining double-stranded DNA. DOXO-mediated stabilization of topoisomerase II prevents resealing of DNA breakage and therefore stops the replication process. The failure of the DNA damage response system to repair lead to apoptosis occurrence. Therefore, cancer cells, as fast-growing cells, show greater sensitivity to the resulting DNA damage than normal cells, as a mechanism used by DOXO to eliminate cancer cells. In addition, as a topoisomerase II inhibitor, DOXO-mediated histone eviction from chromatin correlated with deregulated DNA damage response, and DOXO-mediated oxidative stress also contribute to its chemotherapeutic effects in cancer cells [51,52,53]. DOXO is currently the most effective chemotherapeutic drug in the treatment of breast cancer, with a response rate of approximately 35% in metastatic breast cancer [54,55]. However, its clinical efficacy is limited due to several life-threatening adverse effects. Cardiotoxicity is the most significant side effect as dose-limiting toxicity, which can lead to cardiomyopathy, a lethal disease accounting for about 50% mortality upon development in breast cancer patients [56,57]. Other significant side effects include nephrotoxicity, hepatotoxicity, diarrhea, typhlitis, anemia, and nausea [58,59]. Furthermore, the development of DOXO resistance still remains a major challenge and limits the long-term treatment advantages in breast cancer patients, which is correlated with tumor relapse, poor patient prognosis, and survival [60,61,62].

Increasing evidence strongly indicates that combination treatment of CUR and DOXO may have a potential therapeutic role in improving breast cancer therapy. It was found that the CUR–DOXO combination increased the sensitivity of MCF-7 and MDA-MB-231 cells to DOXO, as evidenced by an approximately two-fold reduction in the half-maximal inhibitory concentration (IC50) of DOXO in treated cells [63]. Elevated expression of ATP-binding cassette (ABC) transporters is amongst the most significant mechanism contributing to chemoresistance [64]. ABC subfamily B member 4 (ABCB4), which acts as an efflux pump to limit the intracellular accumulation of drugs, has been shown to be overexpressed in DOXO-resistant breast cancer cells [65,66]. A recent study has demonstrated that the CUR–DOXO treatment reduced the IC50 value of DOXO and enhanced its sensitivity in DOXO-resistant MCF-7 and MDA-MB-231 cells via inhibition of ABCB4 activity. This effect, which is mediated through the inhibition of ATPase activity of ABCB4 without altering its protein expression, leads to increased intracellular levels of DOXO and the reversal of chemoresistance in treated resistant breast cancer cells [66]. Moreover, DOXO may increase the aggressiveness of breast cancer tumors by triggering epithelial–mesenchymal transition (EMT) [67,68]. CUR was also found to downregulate protein levels of DOXO-induced EMT and metastases regulators, including vimentin, β-catenin, p-AKT, p-Smad2, and p-GSK3β, Snail and Twist in TNBC cells. This CUR inhibitory effect on DOXO-induced EMT was mediated through the suppression of TGF-β and PI3K/AKT signaling pathways in treated cells [69]. Furthermore, the findings from the same study have revealed that CUR was able to sensitize TNBC cells to DOXO and enhanced its antiproliferative effect as evidenced by increased apoptosis marker expression, including cleaved PARP and caspase 3 in treated cells [69]. 

Moreover, constitutive activation of NF-κB not only has been reported in breast cancer progression and development but also in resistance to breast cancer therapy mediated via induction of antiapoptotic proteins [70,71]. Besides, DOXO-induced NF-κB activation also causes more resistance to chemotherapy [72,73]. The underlying mechanism of this activation involves degradation of IκB followed by nuclear translocation of p65 NF-κB, its association with p300 histone acetylase, and subsequently transcription of Bcl-2, leading to a protective response in drug-resistant cells [74]. The study investigating combinational therapeutic efficacy via in vitro and in vivo breast cancer models revealed that CUR effectively sensitized DOXO-resistant cancer cells to apoptosis through suppression of the NF-κB pathway. Cotreatment of DOXO with CUR reduced p65NF-κB translocation to the nucleus, inhibited NF-κB-p300 cross-talk, triggering p53-p300 interaction, and consequently activated p53-dependent apoptosis in DOXO-resistant breast cancer models [74]. Furthermore, in vivo findings also demonstrated that CUR improved DOXO-induced systemic toxicity in DOXO-resistant tumor-bearing mice [74]. Another study also reported the ability of CUR to enhance DOXO cytotoxicity against resistant MCF-7 cells with HER2 overexpression, which is mediated through inhibition of NF-κB and HER2 activation [75]. Besides, it has been reported that overexpression of Aurora-A protein, a mitotic serine threonine kinase, plays a vital role in DOXO insensitivity in breast cancer cells which is mediated via upregulation of Akt-NF-κB signaling axis, triggering drug efflux pump, ABCG2, and Pgp1, and ultimately reduce DOXO accumulation in resistant cells [76,77]. It has been shown that 6 h preincubation with CUR followed by DOXO treatment improved the sensitivity of both DOXO-resistant MCF-7 and MCF-7 cells to this drug by 17- and 7.25-fold, respectively, representing the synergistic effect in treated cells. In addition, the findings from this study have also illustrated that CUR reduced the Aurora-A expression, which leads to p53 stabilization, growth arrest, apoptosis induction contributing to reversing DOXO insensitivity, and increasing sensitivity in DOXO-resistant MCF-7 and MCF-7, respectively [76]. DOXO may also change the state of cancer cells and induce cellular senescence resulting in growth arrest and inhibition of proliferation. However, these cells are still metabolically active and can be involved in tumor relapse and chemoresistance development [78,79,80]. A recent study has demonstrated that senescent MCF-7 cells induced by DOXO have shown increased sensitivity to CUR and its apoptosis effect compared with proliferative cells [81].

### 3.2. Curcumin Enhances the Efficacy of Paclitaxel in Breast Cancer Therapy

Paclitaxel (PTX), commercially known as Taxol, is a natural plant-derived chemotherapy drug that was first isolated in 1971 from the bark of the Pacific Yew tree (Taxus brevifolia). PTX, classified in the taxanes group, is an antimitotic anticancer agent which binds to the β-tubulin of microtubules. This binding stabilizes microtubules and blocks their disassembly, which ultimately results in the induction of mitotic arrest. Besides being a mitotic inhibitor, PTX may induce apoptosis through regulation of Bcl-2 family proteins, regulate the expression of certain microRNAs and modulate immune response via regulation of immune cells, chemokines, and cytokines [82,83,84]. PTX is intravenously given as the first-line therapy for patients with early-stage and metastatic breast cancer. However, chemotherapy-induced peripheral neuropathy is the most common PTX-associated side effect with an incidence rate ranging from 57% to 83% in breast cancer patients. Other adverse effects may include joint and muscle pain., hypersensitivity reaction, edema, nausea and vomiting, hair loss, etc. [85,86,87,88,89]. Moreover, PTX drug resistance is another challenge limiting its clinical application in breast cancer therapy [90,91,92].

Studies have reported an improved efficacy of PTX in combination with CUR for inhibiting breast cancer growth through both in vitro and in vivo breast cancer models. It was found that simultaneous treatment of PTX and CUR exerted synergistic growth inhibition and enhanced the apoptotic and antimigratory effect of PTX in MCF-7 cells. The findings have revealed the involvement of ROS generation, Bcl-2 downregulation, and Bax upregulation in apoptotic cell death. The same study also documented that CUR inhibited the PTX-induced EGFR, ERK1/2, and AKT expression in breast cancer cells [93]. Additionally, the PTX–CUR combination showed enhanced suppression of tumor growth in a dose-dependent manner in the breast cancer mouse model [93]. Moreover, a recent study has shown that combined treatment led to the increased apoptotic effect of PTX in MCF-7 and MDA-MB-231 cells mediated via increased caspase 3 activation, PARP cleavage, and loss of membrane integrity. However, this combination differently regulates NF-κB in those breast cancer cells. Although PTX–CUR treatment upregulated NF-κB expression in MCF-7 cells, it reduced PTX-induced NF-κB in MDA-MB-231 cells [94]. Further study has also shown that combined treatment of PTX with CUR led to regulation of various gene expressions in MCF-7 cells, including downregulation of c-Ha-Ras, Rho-A, Bax, p53, Bcl-xL, NF-κB, and CCND1 in treated cells. However, protein expression results illustrated an increase in Bax and a decrease in Bcl-2 in cotreated cells [95]. When the MDA-MB-231 cell line was analyzed, it was found that the CUR–PTX combination increased p53, caspase-3, caspase-8, Bax, and Bid gene expression but reduced Bcl-xL expression in treated cells. Protein expression analysis also showed Bax upregulation and Bcl-2 downregulation in these TNBC cells. These findings revealed that CUR potentiates the apoptotic effects of PTX in treated breast cancer cells [95]. Another report has revealed increased sensitivity of CUR-treated breast cancer cells to PTX-induced cytotoxicity. The results indicated that MDA-MB-231 cells were more sensitive than MCF-7 cells representing a 4.3- and 3.5-fold increase in PTX efficacy in combination treatment, respectively. Cotreatment-induced sensitivity was mediated through enhanced apoptotic potential as evidenced by the increased cytochrome c, caspase-3, and caspase-8 expression in treated cells. In addition, in vivo study results have demonstrated that PTX–CUR combination exhibited better efficacy in reducing mice mammary tumor size due to suppression of certain molecular markers, including protein kinase C, telomerase, NF-κB, and histone deacetylase in breast cancer mouse model [96].

Besides, the effect of CUR on the reversal of PTX chemoresistance has been identified in breast cancer cells. In one study, the PTX–CUR combination reversed drug resistance and reduced IC50 value from 14.9 µg/mL to 9.4 µg/mL in PTX resistant MCF-7 cells [97]. Further evidence has shown that CUR suppressed the PTX-induced NF-κB activation via inhibition of IκBα kinase activation, IκBα phosphorylation, and degradation in MDA-MB-435 and MDA-MB-231 cells [98,99]. CUR also downregulated PTX-induced expression of proliferative (cyclin D1, c-Myc, Cox-2), antiapoptotic (Bcl-2, Bcl-xL IAP-1, IAP-2, XIAP), metastatic and angiogenetic (VEGF, MMP-9, ICAM-1) proteins and enhanced apoptosis [98,99]. Furthermore, when tested in animal models, combination therapy of PTX and CUR significantly suppressed tumor growth, reduced tumor size, and inhibited lung metastasis in the breast cancer murine model [98,99]. Additionally, cancer stem cells (CSCs) and overexpression of multidrug resistance complex-1 (MDR-1 or P-glycoprotein-1) play a critical role in PTX chemoresistance. P-glycoprotein 1 (Pgp-1), also known as ATP-binding cassette subfamily B member 1, functions as a transmembrane efflux pump involved in the chemotherapy drug uptake and its efflux. Overexpression of aldehyde dehydrogenase-1 (ALDH-1) in breast cancer was also found to be correlated with the stemness features of CSCs [100,101,102]. The findings from a recent report have suggested that cotreatment of CUR and PTX inhibited the ALDH-1 and PTX-induced Pgp-1 expression in MCF-7 cells. This study has also demonstrated the synergistic cytotoxic interaction of CUR–PTX combination accompanied by upregulation of Bax, caspase-7, and caspase-9 along with downregulation of Bcl-2 expression in treated cells. Besides, in in vivo animal experiments on Ehrlich ascites carcinoma (EAC), tumor-bearing mice also showed a reduction in tumor size and marked inhibition of PTX-induced Pgp-1 and -ALDH-1 protein expression (31.55% and 42.01% decrease, respectively) in tumor tissue [103]. Furthermore, a phase II clinical trial consisting of 150 women has investigated the efficacy and safety of intravenous CUR infusion in combination with PTX in advanced and metastatic breast cancer patients [104]. In this trial, either 80 mg/m^2^ of PTX plus placebo or PTX plus 300 mg solution of CUR was intravenously given to the patients once weekly for twelve weeks with three months of follow-up. The obtained data revealed that, in comparison with PTX monotherapy, PTX–CUR combination showed a superior effect on breast cancer patients with respect to physical performance and objective response rate after three months of treatment and short-term follow-up. Furthermore, there was a significant improvement in the levels of carcinoembryonic antigen in the patients’ blood at the end of the treatment and three months of follow-up. The patient’s self-assessed overall performance status also suggested better survival in the curcumin group than the placebo group. Additionally, side effect assessment demonstrated that intravenously administered CUR led to no major safety issues and no reduction in quality of life, but it was beneficial in reducing fatigue [104].

### 3.3. Curcumin Enhances the Efficacy of 5-Fluorouracil in Breast Cancer Therapy

5-fluorouracil (5FU) is a pyrimidine analog of nucleobase uracil in which a fluorine atom presents at the C-5 position in place of hydrogen, and it was first discovered in the late 1950′s. 5FU is an anticancer, antimetabolite agent which is converted to several active metabolites upon entering the cells. These active metabolites inhibited cancer cell proliferation by interfering with DNA synthesis by suppressing nucleotide synthetic enzyme thymidylate synthase activity and consequently inhibiting thymidine formation. Therefore, 5FU is acting as a cell cycle inhibitor, especially on the S phase of cell cycle progression. In addition, it is incorporated in RNA instead of uracil nucleotide and therefore inhibits RNA transcription, which is required for protein and cellular enzyme synthesis. Moreover, the potentiation of apoptosis is another underlying mechanism of its cytotoxic action [105,106,107,108]. Although 5FU has been clinically used as a single agent and most often in combination with other chemotherapies in the management of breast cancer, it causes long-term side effects of cognitive impairment, also known as chemo fog, in breast cancer patients. In addition to cardiotoxicity, hepatotoxicity, nephrotoxicity, and severe myelotoxicity or bone marrow suppression, as major causes of morbidity and mortality, 5FU-induced epithelial ulceration in the digestive system, nausea, vomiting, and diarrhea are also common clinical side effects limiting its efficacy in 5Fu-treated breast cancer patients [109,110,111,112]. The development of drug resistance to 5FU also attenuates its therapeutic benefit in breast cancer therapy. In addition, due to the complexity of 5FU metabolism, mechanisms of acquired drug resistance are also complicated [113,114,115] 

Potentiation of 5FU activity by CUR has been shown in preclinical breast cancer studies. It is well established that thymidylate synthase (TS) overexpression induced by prolonged exposure to 5FU plays a critical role in the development of chemoresistance [115,116,117]. In addition, 5FU-induced NF-κB, AKT, and MAPK pathway activation may lead to resistance in cancer cells [118,119,120]. In this regard, it was found that CUR in combination with 5FU exerted a synergistic cytotoxic effect in various breast cancer cell lines, including MCF-7, MDA-MB-231, SKBR3, and T47D, which are different in terms of receptor status and HER2 expression. This synergistic effect was mediated through apoptosis enhancement as evidenced by increased cleavage of caspase-8, caspase-9, caspase-3, PARP, and DNA fragmentation in co-treated cells with 5FU and CUR [121]. The findings also revealed that CUR sensitizes the breast cancer cells to 5FU through downregulation of IκBα degradation, IKK phosphorylation, TS, and NF-κB expression induced by 5FU in breast cancer cells. It was also shown that TS-dependent downregulation of NF-κB has a decisive role in observed synergism in breast cancer cells [121]. Moreover, CUR inhibited 5FU-activated Akt/PI3K and MAPK pathways and facilitated apoptosis induction in treated cells. Therefore, the chemosensitizing efficacy of CUR was independent of the breast cancer receptor status and was mediated through its regulatory impact on MAPK and Akt as upstream and NF-κB as downstream of TS in breast cancer cells [121]. Furthermore, another study reported that CUR significantly decreased 5FU-induced cytotoxicity while maintaining its antiproliferative activity toward MDA-MB-231 cells. The underlying mechanism of CUR protective function in combined therapy may be attributed to its antioxidant or suppression activity on prosurvival signaling pathways in breast tumors [122].

### 3.4. Curcumin Enhances the Efficacy of Cisplatin in Breast Cancer Therapy

Cisplatin (CIS) is a metal-based anticancer drug that belongs to the platinum-containing group and was first discovered in 1845. CIS mostly enters the cells through active transport, while some molecules are passively diffused through the cell membrane. Inside the cells, CIS loses its chlorine atoms in exchange for the nitrogen atoms of the target guanines, resulting in the formation of platinum-DNA adducts with two consecutive guanine bases within a strand of DNA. In addition to the intrastrand cross-link, CIS can also cause interstrand cross-link in DNA molecules. Therefore, as a DNA-intercalating agent, CIS inhibits DNA replication and transcription, induces DNA damage, and interferes with DNA repair leading to the cell cycle arrest and ultimately apoptotic cell death [123,124]. CIS has been used in breast cancer treatment, and particularly it has recently achieved renewed interest as monotherapy or combined therapy to treat metastatic TNBC patients [125,126,127]. However, CIS-induced organ toxicities are clinically challenging due to the occurrence of nephrotoxicity, neurotoxicity, cardiotoxicity, gastrotoxicity, ototoxicity, myelosuppression, and allergic reactions [124,128]. In addition, although CIS treatment is initially effective in breast cancer therapy, some patients may also show cancer relapses due to the resistance to the CIS regimen. Another obstacle is the high interaction of CIS with plasma proteins which leads to the inactivation of drugs and also nonselective biodistribution. These limitations make the CIS therapy ineffective in breast cancer management [129,130,131].

Numerous studies have reported the potential role of CUR in ameliorating CIS anticancer activity in breast cancer therapy. It was shown that CUR sensitized MCF-7 and MDA-MB-231 to CIS and enhanced its cytotoxicity, as shown by the approximately two-fold reduction in its IC50 in treated cells [63]. Flap endonuclease 1 (FEN1), a member of the structure-specific nuclease family, participating in DNA synthesis and repair processes plays a critical role in preserving genome stability and integrity. FEN1 overexpression has been confirmed to promote rapid proliferation and CIS drug resistance of breast cancer cells. It is also a significant biomarker of lymph node metastasis and poor prognosis in TNBC patients [132,133,134]. It was shown that the combination of CUR with CIS enhanced the sensitivity of MCF-7, MDA-MB-231, and CIS-resistant MCF-7 (MCF-7/DDP) cells to CIS through downregulation of FEN1 and increased apoptosis in treated cells. The findings also suggested that CUR-induced inhibition of ERK phosphorylation is involved in its chemosensitizing effect via targeting FEN1 [135]. In addition, tumor growth inhibition following the combination therapy was mediated through downregulation of CIS-induced FEN1 in a nude mouse xenograft model [135]. Further in vivo study revealed that combined therapy with CUR and CIS inhibited the mammary tumor growth accompanied by increased peroxisome proliferator-activated receptor gamma (PPAR-γ) expression and decreased brain-derived neurotrophic factor (BDNF) expression in mammary tumors [136]. The subsequent finding of this study is that CUR reduced CIS-induced nephrotoxicity, which was mediated through the suppression of proinflammatory cytokines including TNF-α, IL-6, IL-8, and increased anti-inflammatory cytokine IL-10 in mammary tumor-bearing rats [136]. Moreover, autophagy is a natural cellular process involving self-degradation and recycling of cellular components, and induction of autophagy may increase chemosensitivity to the cytotoxic drug [137,138,139]. In addition, colon cancer-associated transcript 1 (CCAT1) is a long noncoding RNA that is involved in tumorigenesis and drug resistance [140,141]. Recent data have illustrated that cotreatment of CUR and CIS could sensitize MCF-7 and MCF-7/DDP cells to CIS through the autophagy activation in breast cancer cells. The underlying mechanism of CUR-increased chemosensitivity was correlated with downregulation of CCAT1 expression and inactivation of PI3K/Akt/mTOR pathway in treated breast cancer cells [142]. Moreover, MCF-7/DDP breast cancer xenografts in nude mice model also confirmed that CUR could activate autophagy to resensitize resistant breast cancer tumors to CIS [142]. Furthermore, another study has indicated that although co-administration of CUR and CIS increased the survival rate of MCF-7 cells, this combinational therapy enhanced the cytotoxicity of CIS in MDA-MB-231 cells and suppression of Aurora-A protein expression and kinase activity, increased apoptosis induction and cell cycle arrest at the sub-G1 and G2/M phases were found to be involved [143].

## 4. Conclusions

**Figure 2:**
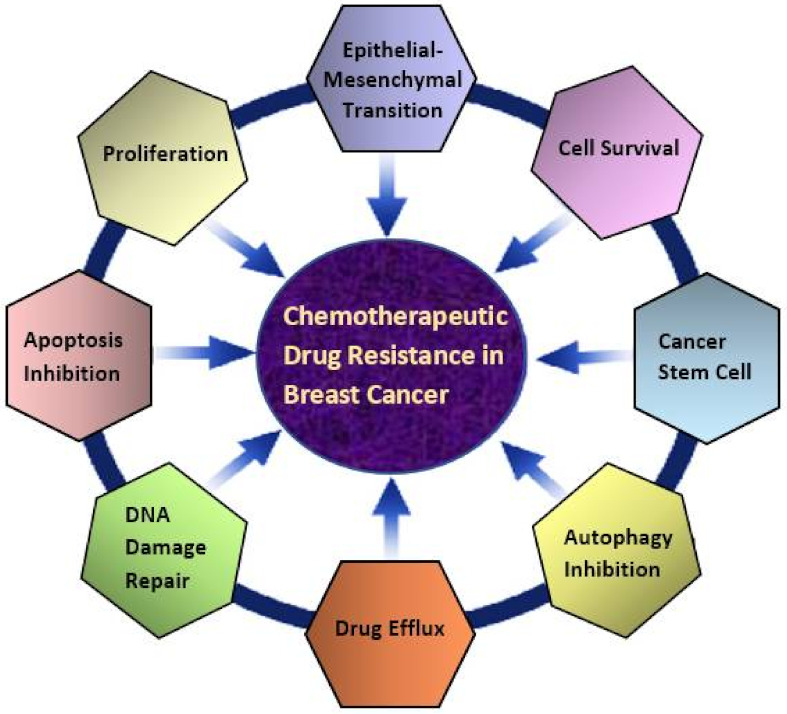
Chemotherapeutic drug resistance in breast cancer. Diverse mechanisms are involved in the development of doxorubicin, paclitaxel, 5-fluorouracil, cisplatin resistance in breast cancer therapy.

Overall, the current review describes the development and progress of curcumin in combination with doxorubicin, paclitaxel, 5-fluorouracil, and cisplatin in breast cancer therapy. These drugs, as the representatives of antitumor antibiotic, antimitotic, antimetabolic, and alkylating agent categories, respectively, are the most widely used chemotherapies for clinical management and treatment in breast cancer patients. However, reported studies in this review have shown various mechanisms contributing to chemoresistance in breast cancer therapy, as illustrated in Figure 2. This study expands the knowledge and detailed understanding of the therapeutic potential and underlying mechanism of curcumin combination chemotherapy. The gathered information highlights the unique role of curcumin in enhancing the anticancer efficacy of conventional anticancer agents via reversing chemoresistance, sensitizing breast cancer cells to drugs, and allowing a significant dose reduction in breast cancer studies. Different molecular targets are modulated by curcumin combination chemotherapy. The regulation of cell cycle (cyclin D1, c-Myc, COX-2, TS, FEN1, HDAC, telomerase), apoptosis (caspases, Bcl-2 family, PARP, ROS, cytochrome c, IAPs, P53), autophagy (CCAT1), and metastases regulators (VEGF, MMP-9, ICAM-1, vimentin, β-catenin, Smad2, GSK3β, Snail and Twist) accompanied by modulation of drug efflux proteins (ABCB4, Pgp-1, Aurora A), kinases (ERK1/2, AKT, IκB, IKK, PKC, PI3k, mTOR), receptors (HER2, EGFR), and transcription of growth factors (TGF-β, NF-kB, Stat3, PPAR-γ, BDNF) have been involved in potentiation of chemotherapeutic activities by curcumin and overcoming drug resistance in in vitro and in vivo studies (Table 1). Moreover, ameliorating chemotherapy-induced side effects following combinational therapy in mammary tumor-bearing animal models also raise the hope for a brighter future of curcumin combined chemotherapy in breast cancer treatment. Collectively, curcumin in combination with chemotherapy drugs may improve their clinical application in breast cancer therapy, and it is of considerable value to develop as an adjunct for combination chemotherapy with current drugs to treat breast cancer. Nonetheless, the current evidence has been mainly obtained from breast cancer preclinical models. Henceforth, clinical trials are warranted to further confirm the chemosensitizing efficacy of curcumin and its prevention role on chemotherapy-induced toxicity in breast cancer patients. Moreover, due to the curcumin’s instability and low bioavailability, the development of nanoformulation and the nano-based codelivery system has been increasingly getting attention as a promising approach in curcumin combination chemotherapy for the more effective breast cancer treatment.

## Figures and Tables

**Table 1:** Combination treatment of curcumin and chemotherapy drugs (doxorubicin, paclitaxel, 5-fluorouracil, and cisplatin) in breast cancer therapy preclinical studies.

Combination Therapy	Cell line/Animal BC Model	Effect/Mechanism	References
CUR+DOXO	MCF-7 MDA-MB-231	•Increased the sensitivity of BC cells to DOXO	[63]
CUR+DOXO	DOXO-resistant MCF-7 DOXO-resistant MDA-MB-231	•Enhanced the sensitivity of BC cells to DOXO •Inhibited ABCB4 activity •Increased intracellular levels of DOXO and reversed chemoresistance	[66]
CUR+DOXO	BT-20	•Suppressed the DOXO-induced TGF-β and PI3K/AKT signaling pathway •Downregulated DOXO-induced vimentin, β-catenin, p-AKT,p-Smad2, and p-GSK3β, Snail and Twist •Upregulated cleaved PARP and caspase 3 •Sensitized TNBC cells to DOXO and enhanced its antiproliferative effect	[69]
CUR+DOXO	DOXO-resistant EAC cells and their derived tumor-bearing mice	•Reduced p65NF-κB translocation to the nucleus and suppressed NF-κB pathway •Activated P53-dependent apoptosis •Sensitized DOXO-resistant cancer cells to apoptosis •Reverted drug resistance and provided survival advantage of doxorubicin-treated tumor-bearing mice	[74]
CUR+DOXO	DOXO-resistant MCF-7 with HER2 overexpression	•Enhanced sensitivity of resistant BC Cells to DOXO via inhibition of HER2 and NF-kB activation	[75]
CUR+DOXO	MCF-7 DOXO-resistant MCF-7	•Reduced the Aurora-A expression •Triggered P53 stabilization •Growth arrest and apoptosis induction •Reversed DOXO insensitivity and increased sensitivity in DOXO-resistant MCF-7 and MCF-7, respectively.	[76]
CUR+PTX	MCF-7 cells S180 cell derived-tumor bearing mice	•ROS generation •Bcl-2 downregulation and Bax upregulation •Inhibited the PTX-induced EGFR, ERK1/2, and AKT expression •Synergistic growth inhibition and enhance the apoptotic and anti-migratory effect of PTX in MCF-7 cells •A marked growth inhibition of the tumor in a dose-dependent manner in breast cancer mouse model	[93]
CUR+PTX	MCF-7 MDA-MB-231	•Increased caspase 3 activation, PARP cleavage, loss of membrane integrity in BC cells •Increased apoptotic effect of PTX in BC cells •Reduced PTX-induced NF-κB in MDA-MB-231 cells	[94]
CUR+PTX	MCF-7 MDA-MB-231	•Down regulation of c-Ha-Ras, Rho-A, p53, Bcl-xL, NF-κB, and CCND1 gene expression in MCF-7 •Upregulation of p53, caspase-3,-8, Bid and downregulation of Bcl-xL gene expression in MDA-MB-231 •Increased Bax and decreased Bcl-2 protein expression in BC cells •Potentiated the apoptosis	[95]
CUR+PTX	MCF-7 MDA-MB-231 DMBA-induced mammary tumor model	•Increased cytochrome c, caspase-3,-8 expression in BC cells •Enhanced apoptotic potential in BC cells •Suppression of protein kinase C, telomerase, NF-κB, and histone deacetylase in breast cancer mouse model •Exhibited better efficacy in reducing mice mammary tumor	[96]
CUR+PTX	MCF-7/ADR	•Reversed drug resistance in PTX resistant MCF-7 cells	[97]
CUR+PTX	MDA-MB-435 MDA-MB-435LVB-derived tumor-bearing mice	•Inhibition of IκBα kinase activation, IκBα phosphorylation, and degradation. •Suppressed the PTX-induced NF-κB activation •Downregulated PTX-induced expression of proliferative (cyclin D1, c-Myc, Cox-2), antiapoptotic (Bcl-2, Bcl-xL IAP-1, IAP-2, XIAP), metastatic and angiogenetic (VEGF, MMP-9, ICAM-1) proteins •Enhanced apoptosis and reversed PTX chemoresistance in BC cells •Decreased the incidence of breast cancer metastasis to the lung and suppressed the expression of NF-κB, Cox-2, and matrix MMP-9 in the animal model. •Suppressed tumor growth, reduced tumor size, and inhibited lung metastasis in breast cancer murine model	[98]
CUR+PTX	MDA-MB-231 cells and their derived tumor-bearing mice	•Suppressed the PTX-Induced NF-κB in BC and potentiated its growth inhibitory effect •Reduced tumor size and decreased tumor cell proliferation, increased apoptosis, and decreased the expression of MMP-9 in the animal model	[99]
CUR+PTX	MCF-7EAC-tumor bearingmice	•Inhibited the ALDH-1 and PTX-induced Pgp-1 expression •Synergistic cytotoxic interaction via upregulation of Bax, caspase-7, -9 and downregulation of Bcl-2 expression •Reduction in tumor size and marked inhibition of PTX-induced Pgp-1, and -ALDH-1 expression in the animal model	[103]
CUR+5FU	MCF-7 MDA-MB-231 SKBR3 T47D	•Exerted synergism effect, which was independent of the receptor status. •Apoptosis enhancement via increased cleavage of caspase-3,-8,-9, PARP and DNA fragmentation •Resensitized the BC cells to 5FU via downregulation of IκBα degradation, IKK phosphorylation, TS, and NF-κB expression induced by 5FU •Inhibited 5FU-activated Akt/PI3K and MAPK pathways and facilitated apoptosis induction	[121]
CUR+CIS	MCF-7MDA-MB-231	•Sensitized BC cells to CIS and enhanced its cytotoxicity as shown by the approximately two-fold reduction in its IC50 in treated cells	[63]
CUR+CIS	MCF-7 MDA-MB-231 MCF-7/DDP MCF-7-derived tumor-bearing mice	•Inhibition of ERK phosphorylation, downregulation of FEN1, and increased apoptosis in BC cells •Enhanced the sensitivity of BC cells to CIS and overcome chemoresistance •Tumor growth inhibition via downregulation of CIS-induced FEN1 in in vivo model	[135]
CUR+CIS	DMBA-induced mammary tumor model	•Inhibited the mammary tumor growth accompanied by increased PPAR-γ and decreased BDNF expression in mammary tumors •Reduced CIS-induced nephrotoxicity via suppression of proinflammatory cytokines (TNF-α, IL-6, IL-8) and increased anti-inflammatory cytokine IL-10 in in vivo model	[136]
CUR+CIS	MCF-7/DDP cellsand their derived tumor-bearing mice	•Inhibited the mammary tumor growth accompanied by increased PPAR-γ and decreased BDNF expression in mammary tumors •Reduced CIS-induced nephrotoxicity via suppression of proinflammatory cytokines (TNF-α, IL-6, IL-8) and increased anti-inflammatory cytokine IL-10 in in vivo model	[142]
CUR+CIS	MDA-MB-231	•Resensitized resistant BC to cisplatin via inducing autophagy by decreasing CCAT1 expression and inactivation of PI3K/Akt/mTOR	[143]

CUR+CIS: curcumin+cisplatin, CUR+DOXO: curcumin+doxorubicin, CUR+PTX: curcumin+paclitaxel, CUR+5FU: curcumin+5-fluorouracil.
